# Self-medication practice among pregnant women in Wolaita Zone, Southern Ethiopia: An institutionally based cross-sectional study

**DOI:** 10.1016/j.heliyon.2023.e13833

**Published:** 2023-02-18

**Authors:** Temesgen Leka Lerango, Amsalu Alagaw, Abayneh Tunje, Eshetu Andarge, Bereket Duko, Asres Bedaso Tilahune, Semalgn Leka Lerango

**Affiliations:** a School of Public Health, College of Medicine and Health Sciences, Dilla University, Dilla, Ethiopia; b School of Public Health, College of Medicine and Health Sciences, Arba Minch University, Arba Minch, Ethiopia; c Flinders Health and Medical Research Institute, College of Medicine and Public Health, Flinders University, Adelaide, South Australia, Australia; d Research Fellow, Australian Centre for Precision Health, UniSA Clinical and Health Sciences, University of South Australia, Adelaide, Australia; e Adjunct Research Fellow, Curtin School of Population Health, Curtin University, Perth, Australia; f University of Technology Sydney, School of Public Health, Sydney, Australia; g School of Medicine, College of Medicine and Health Sciences, Addis Ababa University, Addis Ababa, Ethiopia

**Keywords:** Self-medication, Pregnant women, Wolaita, Southern Ethiopia, Cross-sectional, ANC, Antenatal Care, AOR, Adjusted Odds Ratio, CHI, Community-Based Health Insurance, CI, Confidence Interval, COR, Crude Odds Ratio, ETB, Ethiopian Birr, EFDA, Ethiopian Food and Drug Authority, HC, Health Center, HDA, Health (Women) Development Army, HMIS, Health ManagementInformation System, OTC, Over-The-Counter Medicines, PH, Primary Hospital, POM, Prescription-Only-Medicines, SNNPR, Southern Nations Nationalities and Peoples Region, WHO, World Health Organization

## Abstract

**Background:**

Self-medication is a treatment based on symptoms without prescription and medical consultation. Despite being one of the critical practices that impose a harmful effect on the fetus and the woman herself, evidence on its practice and associated factors are not well-documented. This study, therefore, assessed the self-medication practice and associated factors among pregnant women in Wolaita Zone, Southern Ethiopia.

**Methods:**

An institutionally based cross-sectional study was conducted at public health institutions in Wolaita Zone, Southern Ethiopia by recruiting a total of 408 pregnant women using a systematic random sampling technique between March 2019 and April 2019. We used the Antenatal care (ANC) registry as a sampling frame. A pre-tested, structured, interviewer-administered questionnaire used to depict Self-medication practice and associated factors. Data entered using Epi-data and analyzed by SPSS 23.0.

**Results:**

The overall prevalence of self-medication was 14.9% (95% CI:11–18). The odds of using self-medication may decreased by 75% for women who were in their third trimester (AOR = 0.25, 95% CI: 0.10, 0.64). However, the odds of practicing increased by 13-folds among pregnant women reported earlier (previous) self-medication experience (AOR = 13.62, 95% CI: 6.66–27.84).

**Conclusion:**

The prevalence of self-medication was high in the current study setting. Women's gestational period (third trimester) and earlier self-medication experience were associated with their current self-medication practice.

## 1 Introduction

Self-medication, an element of self-care, is the choice and use of medicines by individuals to treat self-recognized illnesses or symptoms [1]. Self-medication broadly includes old prescriptions, getting medication without a prescription, consulting friends and relatives, and sharing medicines [2,3].

Self-medication is a common practice worldwide with the prevalence varying from country to country for several reasons, but the tendency of self-medication practice has been increasing both in developed and developing countries [[3], [4], [5], [6]]. Literature on self-medication has reported a prevalence of 38.8% among the population of Low and Middle-Income Countries (LMICs) where the prevalence in Sub-Saharan Africa was 40.6% (95% CI: 25.8, 55.8) [4] and 22.9% (95% CI: 9.8, 36) in Ethiopia [7]. Owing to its high prevalence and hazardous effect on the public, self-medication practice is a serious public health concern in low-income countries where resources are scarce [3,7,8].

Rampant irrational use of medicines with no adequate guidance from medical professionals may result in a high probability of inappropriate, incorrect therapy, missed diagnosis, delays in appropriate treatment, microbial resistance, and higher morbidity and death imposing a major global health challenge with significant implications for patients, healthcare systems and communities as a whole [9,10].

Such practice is also common among pregnant women and has gotten serious attention for its erroneous consequences on the health of both mother and the fetus [[11], [12], [13], [14]]. One in five (20%) pregnant women practices self-medication [7]. Evidence also suggests that any pharmaceutical drug use during pregnancy must consider the potential benefits to the mother and the potential risks to the fetus and it should be assisted with critical medical advice [7]. Multi-dimensional underlying and/or contributing factors for self-medication have been documented such as socioeconomic and demographic factors, lifestyle, ready access to drugs, previous (past) experience, public health and environmental factors, and greater availability of medicinal products [3,15].

For reasons of either not being well-known or given less priority among policymakers and program designers, self-medication practice during pregnancy, and possible interventions are not articulated in the current strategies on maternal and reproductive health including antenatal care programs [11]. Therefore, this study aimed to investigate the magnitude of self-medication practice and associated factors among pregnant women in Wolaita Zone, Southern Ethiopia. This study will give baseline data for program designers to develop strategies or guidelines to address the risks of self-medication and its interventions in the antenatal care package.

## 2 Methods

### 2.1 Study setting, design, and population

We conducted an institution-based cross-sectional study from March 11 to Apr. 26, 2019, at public health facilities in Wolaita zone. Wolaita Zone is one of the thirteen zonal administrations of Southern Nations, Nationalities, and People's Region (SNNPR) of Ethiopia, found three hundred kilometers south of the capital city, Addis Ababa. The administrative center of Wolaita Zone is Wolaita https://en.wikipedia.org/wiki/SodoSodo. Currently, there is one teaching referral hospital (Wolaita Sodo University Teaching and Referral Hospital (WUSTRH)), 4 Primary Hospitals(PH), and 69 Health Centers(HCs) in Wolaita Zone. The target populations of the study were all pregnant women who visited the antenatal care unit at public health facilities in Wolaita zone during the study period. The following public health institutions were selected for this study: Areka Health Center, Bale Primary Hospital, Bittena PH, Boditi HC, Sodo HC, and WUSTRH. Women were included in the study irrespective of their gestational age and the pregnant women who were seriously ill and who lived for less than 6 months in the study area at the time of the study period were excluded from the study.

### 2.2 Sampling procedure

The largest sample size for this study was obtained by using single population proportion formula by the consideration of the prevalence of 20.1% prevalence of self-medication practice among pregnant women attending antenatal care in Jima University Specialized Hospital, South West Ethiopia [16], 95% confidence level, 5% degree of precision and Z-value at 95% confidence level of 1.96. The sample size calculated with the above considerations was 247 and with further consideration of a 10% non-response rate, and design effect of 1.5, the final sample size obtained for the study was 408. The samples were obtained using the lists of public health facilities in Wolaita Zonal health bureau. First, six health facilities were selected by lottery method and the number of pregnant women expected to attend each facility was obtained from the last sixth months of antenatal records. Then, a proportionate allocation method was used to get the number of women to be interviewed from each health facility. Finally, a systematic random sampling technique was applied to recruit 408 study participant pregnant women attending antenatal care in selected public health facilities.

### 2.3 Definitions and measurements

**Self-medication –** is a situation in which no health professionals take part in any stage of the therapeutic decision [17]. In this study, self-medication is defined as treatment with oral allopathic (modern) medications, based on symptoms, without any prescription, and without medical consultation during the current pregnancy.

**Prescription Only Medicines (POMs) -** restricted medicinal agents, obtained on prescription, from a designated practitioner; not available for over-the-counter purchase [18].

**Over-The-Counter drugs (OTCDs) -** non-prescription medicines that you can buy without a prescription [19].

**Allopathic medicine -** A system in which medical doctors and other healthcare professionals (such as nurses, pharmacists, and therapists) treat symptoms and diseases using drugs, radiation, or surgery. Also called conventional medicine, biomedicine, mainstream medicine, orthodox medicine, and Western medicine [20].

### 2.4 Data collection instrument and procedures

Data was collected via face-to-face interviews. The questionnaire was developed by reviewing different literature on similar or related topics. The validity of the questionnaire is established by the subject experts which comprised of a pharmacist who had prior research experience, a gynecologist and obstetrician, and an experienced midwife in ANC services. After reviewing all the contents of the questionnaire, they concluded it measures the outcome of interest (self-medication). The questionnaire was translated into Amharic and ‘Wolaittaato doona’ and then back into the English language by language experts to check for its original meaning. Six clinical nurses who had earlier experience in data collection were selected and assigned as data collectors while two clinical pharmacists were assigned as supervisors. A pre-test was conducted on 5% of the actual sample size in Bedessa health center a week before the actual data collection. Based on the findings of the pre-test, proper amendments were made to the questionnaire. Thorough training was given to data collectors and supervisors by the principal investigator. The collected data was sent on a regular basis to supervisors and was checked for completeness, consistency on daily basis, and possible corrections were made by tracing respondents by their address.

### 2.5 Data processing and analysis

Data was checked for completeness and consistency before data entry. Then, data were coded and entered into EpiData 4.4 and exported to SPSS version 23 for analysis. Descriptive statistics such as frequencies, percentages, summary measures, tables, and graphs were used to describe respondents' results. Bivariable logistic regression analysis was done to assess the relationship between each independent variable with self-medication practice and those variables with p-value <0.25 were selected as candidates for multivariate logistic regression analysis. In a multivariate analysis using a backward logistic regression model, an adjusted odds ratio (AOR) with 95% CI was estimated and an independent variable with a p-value <0.05 is considered statistically significant and an independent predictor of self-medication practice.

**2.6 Ethical approval and consent to participate**

Ethical approval was obtained from the Institutional Review Board of Arba Minch University, College of Medicine and Health Sciences. An official support letter was also obtained from Arba Minch University and Wolaita Zone health bureau for undertaking the study. The purpose of the study was explained to all study participants and they were informed that they have full right not to participate and written informed consent was obtained from each study participants. Confidentiality was kept at all levels of the study.

## 3 Results

### 3.1 Sample characteristics of the respondents

Among the 408 samples of pregnant women, 396 pregnant women took part in the study making the response rate 97.1%. The mean (±SD) age of respondents was 24.9(±4.7) years (Table 1).

**Table 1:** Socio-demographic and obstetric characteristics of pregnant women attending ANC at public health facilities in Wolaita Zone, Mar. 11 – Apr. 26, 2019 (n = 396).

Variables		Frequency (N)	Percent (%)
Age	15–23 24–29 >29	172 154 70	43.4 38.9 17.7
Religion	Protestant Orthodox Muslim Others	251 122 19 4	63.4 30.8 4.8 1.0
Ethnicity	Wolaita Amhara Gamo Guraghe Others	340 20 15 10 11	85.8 5.1 3.8 2.5 2.8
Educational status	No formal education Elementary education Sec. education and above	80 139 177	20.2 35.1 44.7
Marital status	Single Married Divorced Widowed	3 385 7 1	0.8 97.2 1.8 0.2
Place of residence	Rural Urban	132 264	33.3 66.7
Had information on self-medication	No Yes	158 238	39.9 60.1
Gestational period (stage of pregnancy)	First trimester Second trimester Third trimester	68 176 152	17.2 44.4 38.4
Average monthly income (in ETB)	<1380 1381–6900 6901–13800	142 248 6	35.9 62.6 1.5

### 3.2 Magnitude and factors associated with self-medication practice

**Fig. 1:**
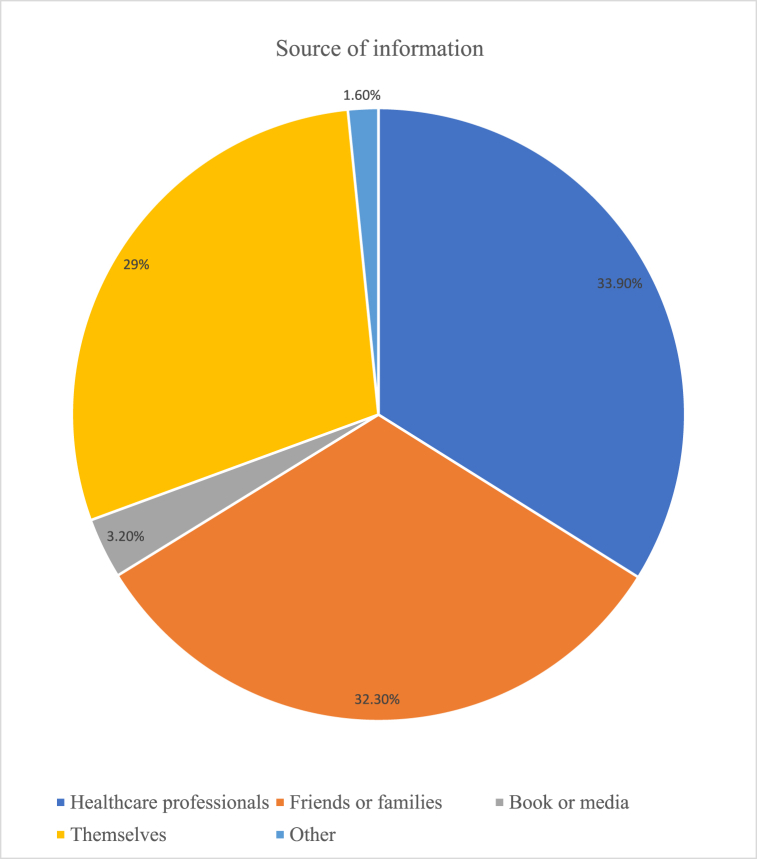
Sources of information for self-medication among pregnant women attending ANC at public health facilities in Wolaita Zone, Mar. 11 – Apr. 26, 2019 (n = 59).

**Fig. 2:**
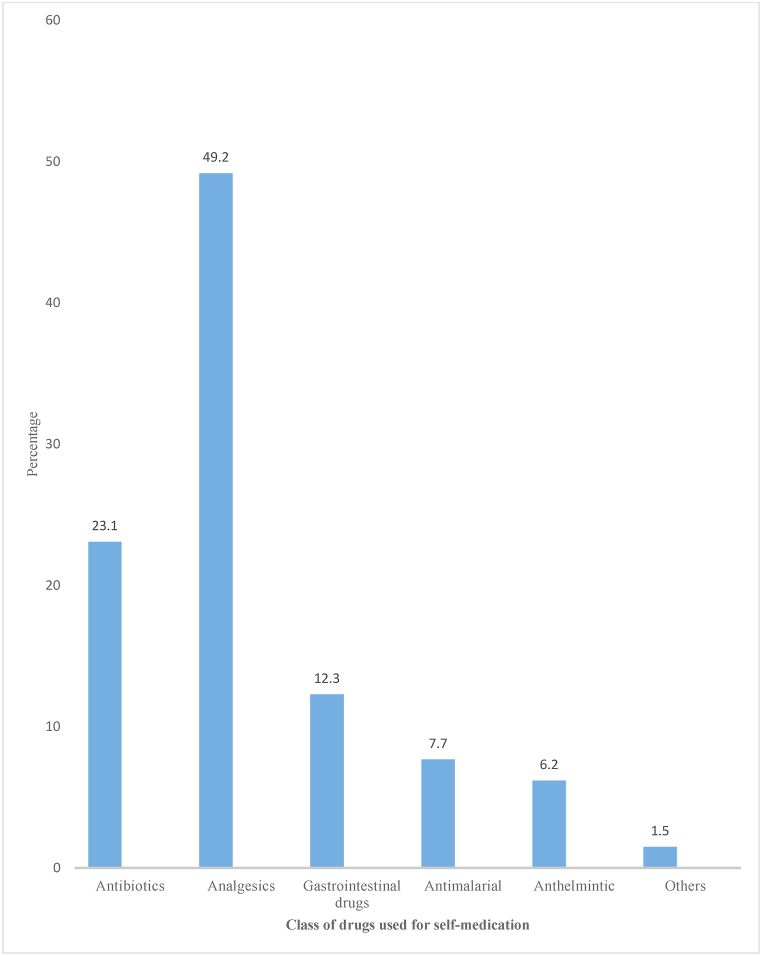
Class of drugs used for self-medication among pregnant women attending ANC at public health facilities in Wolaita Zone, Mar. 11 – Apr. 26, 2019 (n = 59).

Among 396 respondents in the current study, 123(31.1%) have had previous self-medication experience. Concerning self-medication practice during the current pregnancy, the overall prevalence was found to be 14.9% (95% CI: 11%, 18%). The major reason reported to practice self-medication were emergency use 28(44.4%), time-saving 10(15.9%), perceiving illness as minor (not serious) 9(14.3%), and previous self-medication experience 6(9.5%). Women who do not practice self-medication mentioned fear of adverse drug reactions 200(59.3%) and not using the wrong drug 120(35.6%) as their major reasons for not practicing self-medication. The major illnesses/symptoms initiated for self-medication reported were headache 28(43.8%), gastrointestinal disorder (14.1%), and common cold and cough (12.5%). The major source of information was health care professionals (Fig. 1). The most used class of drugs for self-medication in the current study is depicted in (Fig. 2).

The results of bivariable analyses revealed that place of residence, gestational period (stage of pregnancy), membership of health development army, membership of community-based health insurance, and previous self-medication experience were found to be a candidate for multivariable analysis having p-value <0.25 (Table 3).

However, by multivariable analysis, using the backward logistic regression model**,** only women's gestational period and their previous experience of self-medication showed a statistically significant association among the candidate variables selected and fit to the model. Thus, pregnant women in their third trimester were found to be 75% less likely to practice self-medication during the current pregnancy as compared to those pregnant women in their first trimester (AOR = 0.25, 95% CI: 0.10, 0.64), and pregnant women who had previous self-medication experience were found to be 13 times more likely to practice self-medication during the current pregnancy as compared to pregnant women who had no previous self-medication experience (AOR = 13.62, 95% CI: 6.66, 27.84) (Table 2(Table 3).

**Table 2:** Self-medication practice by background characteristics of pregnant women attending ANC at public health facilities in Wolaita Zone, Mar. 11 – Apr. 26, 2019 (n = 396).

Variables	Categories	Self-medication		Chi-square (*X*^*2*^) test	*p-value*
		Yes	No		
Age	15–23 24–29 >29	21 26 12	151 128 58	1.74	0.419
Religion	Protestant Orthodox Muslim Others	32 22 4 1	219 100 15 3	2.75	0.432
Ethnicity	Wolaita Amhara Gamo Guraghe Others	53 3 1 1 1	287 17 14 9 10	1.41	0.842
Educational status	No formal education Elementary education ≥ Sec. education	13 28 18	67 111 159	6.25	0.044
Marital status	Single Married Divorced Widowed	0 58 1 0	3 327 6 1	0.71	0.871
Place of residence	Rural Urban	27 32	105 232	4.82	0.028
Gestational period (trimester)	First Second Third	19 25 15	49 151 137	12.22	0.002
Average monthly income (in ETB)	<1380 1381–6900 6901–13800	21 38 0	121 210 6	1.09	0.581
HDA member	No Yes	22 37	180 157	5.22	0.022
CBHI member	Non-insured Insured	29 30	238 99	10.54	0.001
Had information on SM	No Yes	17 42	141 196	3.55	0.059
Previous SM experience	No Yes	12 47	261 76	76.48	0.000

**Table 3:** Factors associated with self-medication practice among pregnant women attending ANC at public health facilities in Wolaita zone, Mar. 11 – Apr. 26, 2019. (n = 396).

Variables	Categories	Self-medication		Bivariable analyses COR (95% CI)	Multivariable analysis AOR (95% CI)	*p-value*
		Yes	No			
Age	15–23 24–29 >29	21 26 12	151 128 58	1.00 1.46 (0.79, 2.72) 1.49 (0.69, 3.22)	1.00 1.83 (0.86, 3.87) 1.25 (0.48, 3.28)	*0.117* *0.653*
Educational status	No formal educ. Elementary educ. ≥ Sec. educ.	13 28 18	67 111 159	1.00 1.30 (0.63, 2.68) 0.58 (0.27, 1.26)	1.00 2.45 (0.99, 6.03) 0.95 (0.38, 2.37)	*0.052* *0.907*
Place of residence	Rural Urban	27 32	105 232	1.00 0.54 (0.31, 0.94)	1.00 0.60 (0.28, 1.26)	*0.176*
Gestational period (trimester)	First Second Third	19 25 15	49 151 137	1.00 0.43 (0.22, 0.84) 0.28 (0.13, 0.60)	1.00 0.47 (0.21, 1.11) **0.25 (0.10, 0.64)**	*0.061* 0.003*
HDA member	No Yes	22 37	180 157	1.00 1.93 (1.09, 3.41)	1.00 1.78 (0.91, 3.48)	*0.094*
CBHI member	Non-insured Insured	29 30	238 99	1.00 2.49 (1.42, 4.36)	1.00 1.17 (0.56, 2.44)	*0.672*
Had information on SM	No Yes	17 42	141 196	1.00 1.78 (0.97, 3.25)	1.00 1.07 (0.50, 2.29)	*0.871*
Previous SM experience	No Yes	12 47	261 76	1.00 13.45 (6.79, 26.64)	1.00 **13.62 (6.66, 27.84)**	0.000*

(* = a statistically significant variable at p < 0.05 in multivariable logistic regression analysis).

## 4 Discussion

This study found a high magnitude of irrational self-medication practice among pregnant women in Wolaita Zone, Southern Ethiopia. Women's gestational period (third trimester) and previous self-medication experience were factors associated with self-medication practice in the current study setting. Given the dearth of evidence in the Southern region of the country, the findings will contribute to the literature so that interventions can be sought for this dangerous practice particularly early during pregnancy. The findings may also have an input to program planners in the local area and policymakers at large to intervene in the sustained practice of self-medication among the target women in the study area and by extension to the country.

The overall prevalence of self-medication practice was found to be 14.9%. A similar finding was reported from previous studies in Goba-Ethiopia, Congo, Gujarat-India, Mexico, and the Netherlands where self-medication practice was 15.5%, 14%, 8.5%, 21.9%, and 12.5% respectively [14,[21], [22], [23], [24]]. However, it was lower than the findings from Addis Ababa and Harar-Ethiopia, Tanzania, Nigeria, and Iran which were 26.6%, 69.4%, 46.24%, 85%, and 43.5% respectively [11,[25], [26], [27], [28]]. The possible explanation for the differences could be either owing to the present study has assessed only conventional (modern) medicines while some previous studies have assessed the prevalence of self-medication with conventional (modern) and herbal medications. It might be due to the disparities in the health care system and the regulators among the countries and regions.

In the current study, the classes of drugs most utilized were analgesics, antibiotics, and gastrointestinal drugs of which around 50% were gotten from retail drug outlets. This finding revealed a need for adequate regulatory enforcement by the Ethiopian Food and Drug Authority (EFDA) in retail drug outlets where a significant proportion of drugs for self-medication by pregnant women are obtained. Nowadays self-medication practice is one of the leading contributors to global antimicrobial resistance [9], thus irrational dispensing of antimicrobials without a prescription to pregnant women should be prohibited. As revealed in this study gestational period was significantly associated with self-medication practice with lower odds of self-medication practice in the later pregnancy period. The finding implies that irrational use is more common in the earlier trimesters while that was the time when most drugs are contraindicated for fear of their teratogenic effect. The possible reason could be that most physiological changes and gestation-induced illnesses occur in the first trimester of pregnancy, so to get emergency relief for the perceived illnesses in this stage, pregnant women might prefer to use medicines on their own than visit healthcare facilities. In addition, as the gestational period increases the likelihood of pregnant women visiting healthcare facilities increases, and hence getting advice on all necessary cautions to be undertaken during pregnancy. This is supported by the significant number of literatures from Ethiopia that reported late initiation of antenatal care by pregnant women [[29], [30], [31]]. The finding in this study is contrary to a study done in Nigeria [32]. The possible reason for the difference in the findings might be due to the difference in study methods, the study in Nigeria was a prospective cross-sectional exploratory study that had recruited consecutive samples among all pregnant women, and the differences in the populations. While a previous research finding from Nigeria reported the third trimester (AOR = 4.2, 95% CI: 3.1, 5.6) [32] to be associated with self-medication practice that contradicts the finding from the current study, this could indicate a need for further research to investigate the contradicting findings.

Among pregnant women who have self-medication practice in the current study, 47 out of 59(80%) had previous self-medication experience which is in line with a finding from Addis Ababa in which 121 out of 164(74%) had previous self-medication experience [11]. This result should be considered by the policy planners as it could be a worthy input in developing strategies that can be incorporated into the antenatal care package. The result of this study also revealed the presence of a significant association between previous self-medication experience and the current self-medication practice with higher odds among those having previous experience. This is in line with findings reported in previous studies done in Jimma, Addis Ababa, Harar, and DR Congo [11,16,22,25]. The possible reason for the sustained practice might be poor awareness of the adverse effects of drugs on themselves and their fetus. In addition, negligence to one's health safety might lead to the reuse of leftover medications and sharing medications between families and neighbors.

In conclusion, healthcare providers should focus on counseling pregnant women visiting for ANC on self-medication experience prior to the current pregnancy and deliver adequate counseling on self-medication to those who had experience. The government's regulatory agency, the Ethiopian Food and Drug Authority (EFDA), should control sell of both prescription-only medications and over-the-counter medications to society.

### 4.1 Limitations of the study

This study has a few limitations to consider. It assessed the magnitude of self-medication with modern (conventional) medicines; women's herbal medication use was not assessed. In addition to this, self-medication was assessed using a structured questionnaire for self-report which might be affected by social desirability bias that would underestimate the result.

## Author contribution statement

Temesgen Leka Lerango; Amsalu Alagaw; Abayneh Tunje: Conceived and designed the experiments; Performed the experiments; Analyzed and interpreted the data; Contributed reagents, materials, analysis tools or data; Wrote the paper.Eshetu Andarge; Bereket Duko; Asres Bedaso Tilahune; Semalgn Leka Lerango: Conceived and designed the experiments; Analyzed and interpreted the data; Contributed reagents, materials, analysis tools or data; Wrote the paper.

## Funding statement

This research did not receive any specific grant from funding agencies in the public, commercial, or not-for-profit sectors.

## Data availability statement

Data will be made available on request.

Questionnaire (English Version)

Part-01 Socio-demographic information.

**Table:** 

Serial No.	Eligible questions			Possible answers	Skip to
101	Age (in years)			______________	
102	Religion			1. Protestant 2. Orthodox 3. Muslim 4. Others, (please specify) ______________	
103	Ethnicity			1. Wolaita 2. Amhara 3. Gammo 4. Guraghe 5. Others, (please specify) _______________	
104	Educational status			1. No formal education 2. Elementary education 3. Secondary education and above	
105	Marital status			1. Single 2. Divorced 3. Married 4. Widowed	
106	Place of residence			1. Rural 2. Urban	
107	Gestational period pregnancy)	(Stage	of	1. First trimester 2. Second trimester 3. Third trimester	
108	Average monthly income (in ETB)			_____________________	

Part-2. Factors related with self-medication.

**Table:** 

201	Are you a member of Health Development Army?	1. Yes 2. No				
202	Are you a member of Community Based Health Insurance (CBHI)?	1. Yes 2. No				
203	Have you ever heard about self-medication?	1. Yes 2. No				
204	Do you have previous self-medication experience?	1. Yes 2. No				
205	Have you ever self-medicated during current pregnancy?	1. Yes 2. No				
206	If No to 205, why not you practice self-medication?	1. Fear of drug adverse reactions 2. Fear of drug resistance 3. Fear of over treatment 4. Fear of under treatment 5. Not to use wrong drug 6. Others (Please specify) ______________				
207	If Yes to 205, what was your source of information?	1. Healthcare professionals 2. Friends and/or families 3. Book/Media 4. Previous experience 5. Others (please specify) _______________				

208	What was the illness (symptom) that lead you practice self-medication?	1. Headache 2. Fever 3. Cold and cough 4. Morning sickness 5. Urinary tract infection 6. Gastrointestinal disorder 7. Diarrhea 8. Back pain 9. Malaria 10. Others, (please specify) _____________	
209	Where do you get the drugs for self-medication? (Source of drugs)	1. Public health institution 2. Private health institution 3. Retail drug outlet 4. Sharing with neighbors/ families/friends 5. Market and any shop 6. Others, (please specify) ________________	
210	How do you request the drug if the source for the drug is retail drug outlet?	1. By mentioning symptoms 2. By mentioning class of drug (its name) 3. By showing drug container (package) 4. By showing a piece of paper on which, the name of the drug written. 5. Others, (please specify) ______________	

211	What was the medication that you took to alleviate the symptom?	1. Antibiotics 2. Analgesic/antipyretic 3. Gastrointestinal drugs 4. Antimalarial drugs 5. Anthelmintic drugs 6. Others, (please specify) __________________	
212	Could you show me please?	1. Yes, write its name from the package 2. No	
213	Can you please tell me why you practice self-medication?	1. Emergency use 2. Time saving 3. Illness is minor (not serious) 4. Less expensive (Cheaper) 5. Previous experience 6. High cost of health professionals' visits 7. Health facility is unavailable 8. Easy access of medicinal products 9. Families/peer pressure 10. Long waiting time at health facilities 11. Others (please specify) ___________________	
214	Why do you not visit health facilities when you feel sick rather than taking drugs by your own?	1. Cost for health service is high 2. Easy access to medicines from RDOs 3. Lack of trained health professionals 4. Poor ethics of health professionals 5. Being embarrassed to tell about disease 6. Other (please specify) __________________	

## Declaration of competing interest

The authors declare that they have no known competing financial interests or personal relationships that could have appeared to influence the work reported in this paper.
